# *In vitro* metabolic signaling in two intestinal bacterial isolates: glutamate-driven transcriptional and functional reprogramming in *Clostridium butyricum* and *Bacteroides thetaiotaomicron*

**DOI:** 10.1128/msphere.00190-26

**Published:** 2026-08-10

**Authors:** Nazanin Nematzadeh Somehsaraei, Joshua Lemuel Hadi, Mohammadali Khan Mirzaei, Li Deng, Michael Gigl, Corinna Dawid, Philippe Schmitt-Kopplin, Itır Geydirici, Michael Schloter, Silvia Gschwendtner

**Affiliations:** 1 ZIEL-Institute for Food & Health, Technical University of Munich (TUM) 234263 , Freising, Germany; 2 Research Unit for Comparative Microbiome Analysis, Helmholtz Center Munich 84928 , Munich, Germany; 3 Institute of Virology, Helmholtz Center Munich 687160, Neuherberg, Germany; 4 Chair of Prevention of Microbial Diseases, Central Institute of Infection Prevention (ZIP), Technical University of Munich (TUM) 9184, Freising, Germany; 5 Junior Research Group Food Processing and Health, ZIEL-Institute for Food & Health, Technical University of Munich (TUM) 234263, Freising, Germany; 6 Professorship for Chemosensory Food Systems, Technical University of Munich 9184, Freising, Germany; 7 Leibniz Institute for Food Systems Biology, Technical University of Munich (TUM) 9184, Freising, Germany; 8 Research Unit Analytical BioGeoChemistry, Helmholtz Center Munich, Neuherberg, Germany; 9 Chair of Analytical Food Chemistry, Technical University of Munich (TUM) 9184, Freising, Germany; 10 Chair of Environmental Microbiology, Technical University of Munich (TUM) 9184, Freising, Germany; Kansas State University, Manhattan, Kansas, USA

**Keywords:** monosodium glutamate, gut microbiota, transcriptomics, metabolomics, strain-specific responses, butyrate, metabolic health

## Abstract

Monosodium glutamate (MSG; L-glutamate monosodium salt) is among the most widely used flavor enhancers, yet its molecular effects on gut microbial physiology remain poorly understood. Here, we examined the strain-specific transcriptomic and metabolic responses of *Clostridium butyricum* and *Bacteroides thetaiotaomicron* grown in pure anaerobic culture with 0.1% (wt/vol) MSG. Although MSG exposure was not associated with major changes in total bacterial biomass dynamics, both species showed a temporal functional reprogramming. In *C. butyricum*, MSG rapidly intensified metabolic activity to capitalize on exogenous glutamate by activating central carbon metabolism, redox-balancing pathways, and the GABA shunt, thereby collectively enhancing butyrate synthesis, a metabolite associated with gut barrier integrity, energy regulation, and anti-inflammatory signaling. On the other hand, *B. thetaiotaomicron* adopted a conservative, homeostatic response, suppressing glycan utilization and central carbon and energy metabolism, and maintaining stable SCFA production through controlled regulation that buffered against abrupt dietary perturbations. Overall, MSG functioned as a potent metabolic signal, triggering distinct adaptive strategies in two dominant gut bacteria.

**IMPORTANCE:**

The impact of monosodium glutamate (MSG) as a highly consumed food additive on the gut microbiome is often overlooked, and community-level analyses reveal little change, masking distinct phenotypic responses of individual strains. By combining gene expression and metabolite profiling using two key human gut bacteria, we show that MSG is sensed as a metabolic signal. A butyrate-producing gut bacterium increases energy metabolism and butyrate production, and a fiber-degrading gut bacterium transiently moderates metabolism to maintain stable fermentation products. These differences suggest that MSG's physiological effects may depend on which bacterial groups dominate an individual’s microbiome.

## INTRODUCTION

The human gut is a complex and dynamic ecosystem, serving as an interface between dietary inputs and host physiology, and, therefore, plays a crucial role in human health. Even short-term shifts in nutrient intake can cause rapid changes in microbial community structure and function (1, 2), influencing energy balance, immune modulation, and neurochemical signaling (3, 4). However, how specific dietary molecules modulate microbial metabolism, gene expression, and functional pathways remains poorly understood.

Among food-derived compounds, amino acids and their derivatives play an important role because they serve simultaneously as nutrients and signaling molecules (5). One of the most abundant compounds in the human diet is glutamate, comprising up to 10% of naturally occurring amino acids in the diet. Notably, high levels of naturally occurring free glutamate are found in many umami-rich foods, including tomatoes, aged cheeses, mushrooms, and fermented seasonings such as soy or fish sauce (6). Nowadays, glutamate is widely used in the food industry as a flavor enhancer, primarily as monosodium glutamate (MSG; the sodium salt of L-glutamic acid). As a result, MSG daily intake has increased to up to 1.0 g day^−1^ in Western diets and about 1.5–3.0 g day^−1^ in Asia (7). Although distinct MSG effects on human health are not well characterized, high doses of free glutamate synthesized during industrial processes have shown toxic effects on living cells (8, 9) and laboratory animal models, such as kidney disorders (10), neurotoxicity, obesity, insulin signaling disruptions (11), and negative effects on the reproductive organs (12). In addition, higher individual MSG intake has been linked to an increased risk of metabolic syndrome (13), and a reduction of dietary free glutamate improved symptoms in individuals with fibromyalgia and irritable bowel syndrome (14), indicating an association between MSG consumption and metabolic dysbiosis.

Almost 95% of ingested glutamate is extracted and metabolized by the splanchnic bed during first-pass metabolism (15, 16), and the oxidation to CO_2_ extensively via the TCA cycle can generate roughly one-third of the total energy demand of the intestinal mucosa (16, 17). In addition, glutamate serves as a precursor for multiple biosynthetic pathways, such as amino acid biosynthesis, glutathione, and γ-aminobutyric acid (GABA) metabolism (17). Moreover, it plays a crucial role in the enteric nervous system as a signaling molecule and can modulate neuroendocrine reflexes through multiple receptors and transporters spread along the gut (18, 19).

Despite its central metabolic role, the impact of exogenous glutamate on intestinal microorganisms has received only limited attention so far. For bacteria, glutamate serves not only as a carbon and nitrogen source but also as a regulatory molecule modulating acid resistance, osmotic stress, and redox balance. Glutamate decarboxylases convert glutamate to GABA while consuming an intracellular proton during acid stress (20, 21). Glutamate can also act as an early osmoprotective signal by accumulating potassium glutamate after osmotic upshift (22, 23). In addition, glutamate dehydrogenase, together with the GS-GOGAT cycle, links carbon and nitrogen flux to NAD(P)H-dependent redox balance (24). Thus, exogenous MSG may trigger canonical stress-protection pathways or broader metabolic reprogramming depending on species-specific regulatory architecture.

These functions suggest that exogenous MSG could induce distinct transcriptional and metabolic responses across bacterial phyla depending on their ecological roles and metabolic networks. The few studies addressing the consequences of MSG on the gut microbiome mostly focused on shifts in microbial community structure using the 16S rRNA gene as a marker for metabarcoding (25, 26). However, a simple analysis of microbial community structure is not able to differentiate between passive nutrient assimilation and active metabolic reprogramming. Therefore, an integrative multi-omics approach combining transcriptomics and metabolomics is required to understand how MSG reshapes cellular activity and metabolite output.

To address these gaps, we investigated the strain-specific molecular responses of two phylogenetically distinct model gut bacteria selected to represent contrasting functional guilds relevant to MSG-associated metabolism, rather than the full diversity of the gut microbiota. *Clostridium butyricum* was selected as a model for a butyrate producer with reported immunomodulatory properties, and a distinctive capacity to convert lactate and acetate into butyrate (27, 28). *Bacteroides thetaiotaomicron* was selected to serve as a model organism for polysaccharide foraging due to its extensive repertoire of polysaccharide utilization loci (29, 30), as well as for its role in host immune modulation (31). Previous studies have shown that these organisms exhibit fundamentally different growth dynamics and metabolic strategies. *B. thetaiotaomicron* demonstrates the upregulation of genes in the glutamate decarboxylase (GAD) system, associated with GABA production, during the transition from late exponential to early-stationary phase (21). In contrast, *C. butyricum* shifts toward a butyrate-centered metabolic profile that predominates during stationary phase (32). These phase-dependent metabolic distinctions formed the basis for the timing and design of our MSG treatment.

Using longitudinal RNA-seq and targeted metabolomics following MSG exposure under strictly anaerobic conditions, we asked whether transcriptional reprogramming is accompanied by measurable shifts in fermentation products. We hypothesized that MSG acts as both a nutrient and a metabolic signal: in *C. butyricum,* it would increase glutamate-linked energy and redox metabolism and butyrate synthesis, and in *B. thetaiotaomicron,* it would transiently suppress energy-expensive pathways to preserve redox and metabolic homeostasis and might induce the GABA shunt pathway (21). By resolving these strategies, our study provides a molecular framework for understanding how dietary amino acids shape functional differentiation within the gut microbiome.

## MATERIALS AND METHODS

### Bacterial strains and culture conditions

Two bacterial species, *Bacteroides thetaiotaomicron* DSM 2079 (Bacteroidota) and *Clostridium butyricum* DSM 10702 (Bacillota), were used as model organisms representing two distinct functional groups of the human intestinal microbiota. Both strains were obtained from the Deutsche Sammlung von Mikroorganismen und Zellkulturen (DSMZ; Braunschweig, Germany) and stored as 30% (vol/vol) glycerol stocks at −80°C until use.

Cultures were routinely maintained at 37°C without stirring under strict anaerobic conditions. In 250 mL sealed serum bottles, 100 mL brain heart infusion (BHI) medium supplemented with 5 g L^−1^ yeast extract, 0.5 g L^−1^ L-cysteine-HCl, and 5 mg L^−1^ hemin (Sigma-Aldrich Chemie, Holzkirchen, Germany) was added as a common rich anaerobic medium that supports reproducible growth of both phylogenetically distinct strains (33). The pH was adjusted to 7.0 prior to autoclaving to simulate colonic conditions (34, 35). To ensure an oxygen-free environment, media were equilibrated in an anaerobic workstation using an 80:20 (vol/vol) N_2_/CO_2_ gas mixture, and resazurin (5 mg L^−1^; Sigma-Aldrich Chemie, Holzkirchen, Germany) served as a redox indicator.

### Growth-curve experiment

Strain-specific growth dynamics under the defined culture conditions were established prior to MSG exposure; experimental details are provided in the Supplemental material.

### Treatment and experimental design

The main MSG experiment was conducted using 10 independent anaerobic cultures per strain, with 5 serving as untreated controls and 5 assigned to MSG treatment. For the inoculation, similar to the growth-curve experiment, ~10^8^ cells per 100 mL culture were used for both strains. After 18 h of incubation, corresponding to the mid-stationary phase for *C. butyricum* and early-stationary phase for *B. thetaiotaomicron*, determined by growth-curve measures, a sterile, anoxic MSG solution (L-glutamic acid monosodium salt monohydrate; Thermo Fisher Scientific, Dreieich, Germany) was added to the treatment cultures. The timing of MSG addition was chosen following strain-specific metabolic states previously reported for each strain, including GABA-associated activity in *B. thetaiotaomicron* (21) and butyrate-oriented metabolism in *C. butyricum (*32). This approach minimized biomass-accumulation effects while capturing early MSG responses in metabolically remodeled stationary-phase states representing colonic conditions (36). Since direct *in vivo* measurements of ingested free glutamate delivery to the colon are lacking, MSG was added at 0.1% (wt/vol; 5.35 mM) to model an upper-range colonic exposure based on a high-intake scenario of ~12 g MSG (17) and reports that 7%–10% of dietary protein may reach the large intestine (37).

Samples (2 mL) were collected at 0 (immediately prior to treatment), 2, and 5 h post-treatment for transcriptomic analysis to capture early adaptive responses. According to time-resolved multi-omics studies, which have reported a time-lag between transcriptomic and metabolomic changes in bacteria (38–40), we collected samples at 0, 2, 5, and 8 h post-treatment for metabolomic analysis. Following centrifugation (10,000 × *g*, 10 min, 4°C), supernatants and cell pellets were separated and stored at −80°C until further analysis.

### DNA extraction and quantitative real-time PCR

Genomic DNA was isolated from bacterial cultures using the PureLink Genomic DNA Mini Kit (Thermo Fisher Scientific, Darmstadt, Germany) according to the manufacturer’s protocol for Gram-positive bacteria (tested and reliable protocol for both strains). qPCR was performed using a strain-specific TaqMan assay to estimate total genome-equivalent biomass dynamics and was not interpreted as a direct measure of viable cell numbers. OD_600_ measurements from the preliminary growth-curve experiment were used as an independent optical proxy to define the growth phase at which MSG was added. Reaction preparation, target gene, primer, probe sequences, and thermocycling conditions are provided in the Supplemental material.

### RNA extraction and library preparation

Total RNA was extracted from frozen pellets using PureLink RNA Mini Kit (Thermo Fisher Scientific, Dreieich, Germany) according to the manufacturer’s instructions in conjunction with TRIzol reagent (Invitrogen, Thermo Fisher Scientific, Germany). DNase I, amplification grade (Thermo Fisher Scientific, Dreieich, Germany), and RNA Clean & Concentrator-5 Kit (Zymo Research, Bad Homburg, Germany) were used to degrade residual genomic DNA and purify the RNA extract, respectively.

Complementary DNA (cDNA) was synthesized using SuperScript IV VILO Master Mix (Invitrogen, Thermo Fisher Scientific, Germany). Libraries were prepared from cDNA using the NEBNext Ultra II FS DNA Library Prep Kit (New England Biolabs, Frankfurt am Main, Germany), optimized for low-input RNA-seq, following the manufacturer’s instructions. Libraries were normalized to 2 nM, pooled, and sequenced on an Illumina NextSeq 500/550 platform (paired-end 2 × 150 bp; Illumina, San Diego, CA, USA).

### Comparative genome analysis

To compare the genomic potential of the two gut bacteria, we performed a two-genome comparative analysis using anvi’o (V8) (41). Further details are provided in the Supplemental material.

### Transcriptomic data processing and differential expression analysis

Raw reads were demultiplexed using bcl2fastq (v2.20), with quality assessed by FastQC/MultiQC after lane merging, trimming, and rRNA depletion. Paired-end reads were adapter- and quality-filtered with fastp (v0.23.4; *Q* ≥ 20, minimum length 30 bp) (42), and residual rRNA was removed *in silico* using SortMeRNA (v4.3.6) and the SILVA and Rfam databases (43). Functional annotation was performed using Bakta (v1.4.0) (44) and DIAMOND (v2.0.15) (45) with KEGG orthology (46). Pseudo-alignment and quantification were carried out using Kallisto (v0.48.0) (47), and transcript counts were summarized to gene level using Tximport (v1.20.0) (48). All statistical analysis and data visualization were performed in RStudio (v4.5.2) (49). Differential expression was analyzed using DESeq2 (v1.32.0) (50) with a generalized linear model including treatment, time, and interaction terms. Genes with adjusted *P* < 0.05 and |log_2_FC| > 1 were considered significant (51). Principal coordinates analysis (PCoA) based on Euclidean distances of variance-stabilized transcript counts was used to visualize global expression patterns in MSG-treated samples, and the effect of time was tested by permutational multivariate analysis of variance (PERMANOVA; adonis2, 999 permutations) (52). Enrichment of KEGG pathways was evaluated via gene set enrichment analysis (GSEA) using fgsea (v1.24.0) (53). Visualizations were produced with ggplot2 (v3.4.4) (54).

### Metabolite measurement and statistical analysis

Following the 3-NPH derivatization protocol described by reference 55, −80°C stored culture supernatants were targeted for LC-MS/MS analysis (details in Supplemental material). The panel of quantified metabolites included acetate, propionate, butyrate, valerate, caproate, lactate, isobutyrate, 2-methylbutyrate, isovalerate, isocaproate, and glutamate. Metabolite concentrations were determined by stable isotope dilution analysis using calibration standards, and only metabolites consistently detected above the limit of detection were included in downstream analysis.

Log-transformed metabolite concentrations were analyzed using linear mixed-effects models (nlme::lme) with treatment, time, and their interaction as fixed effects and biological replicate as a random effect. Fixed effects were evaluated using type III tests (car::Anova), and *post-hoc* comparisons were performed using Tukey-adjusted estimated marginal means (emmeans v1.8.8) (56). Multivariate SCFA (short-chain fatty acid) profiles were analyzed by Bray-Curtis dissimilarity (57) and PERMANOVA. A two-tailed *P* < 0.05 was considered statistically significant.

## RESULTS

### Growth dynamics

Growth curves based on OD_600_ measurements showed that *C. butyricum* reached the stationary phase after approximately 12 h (OD_600_ = 0.82 ± 0.01) and *B. thetaiotaomicron* after approximately 18 h (OD_600_ = 0.83 ± 0.02) under the applied culture conditions (Fig. S1). MSG was added after 18 h of incubation, corresponding to stationary-phase conditions for both strains. During the subsequent experimental window, MSG treatment was not associated with significant treatment-dependent changes in total bacterial biomass as estimated by strain-specific qPCR (*P* > 0.05, mixed-effects model; Table S2). Together, the OD_600_-based growth-curve data and qPCR-based genome-equivalent biomass estimates support the conclusion that MSG did not cause major biomass accumulation during the experiment. However, as neither OD_600_ nor qPCR directly distinguishes viable from non-viable cells, these data are interpreted as indicators of total biomass dynamics rather than viable cell growth.

### Clostridium butyricum

#### Transcriptional reprogramming

**Fig 1:**
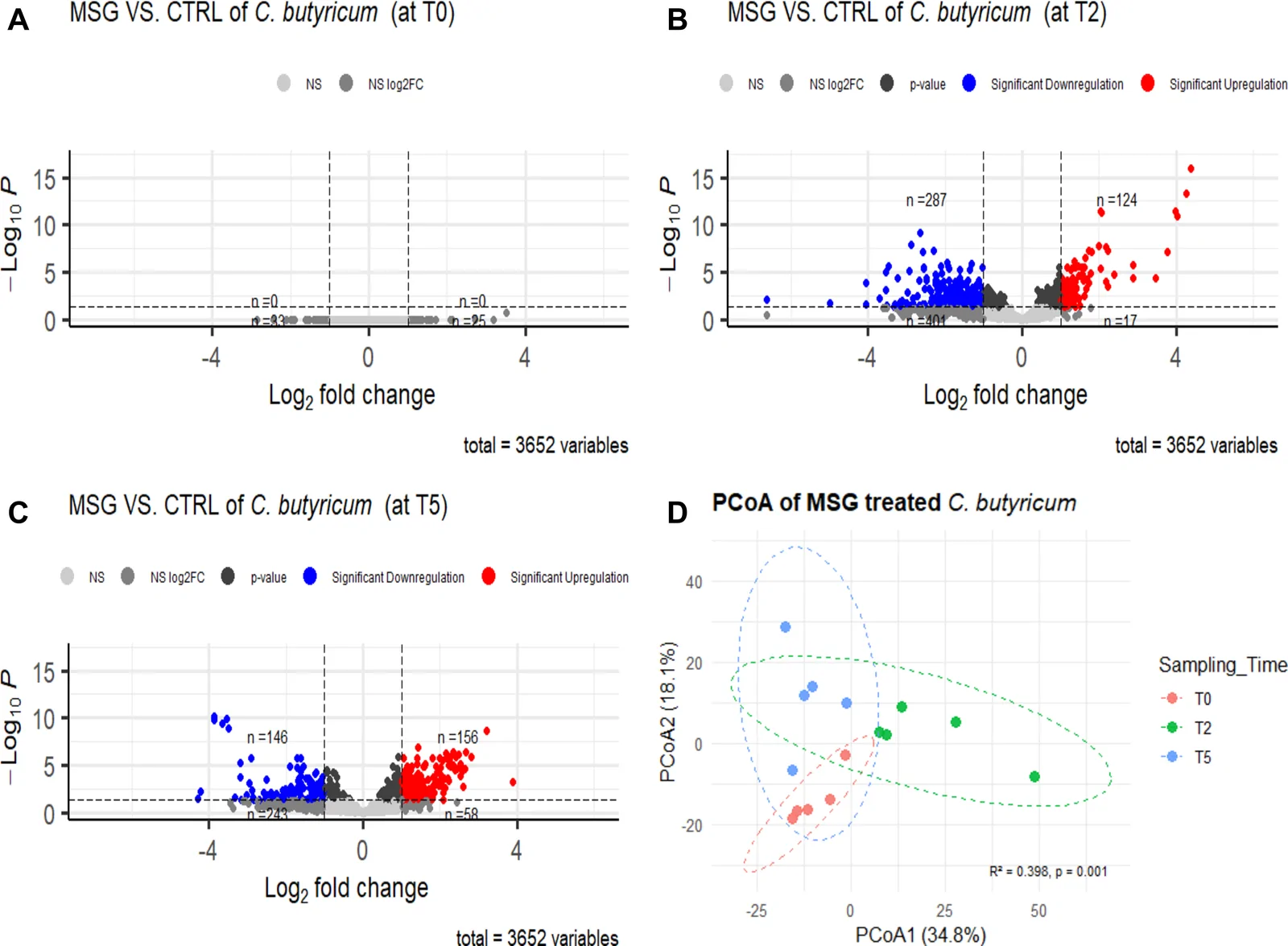
Volcano plots of the differentially expressed genes of *C. butyricum*. MSG-treated versus control at (**A**) baseline (T0), (**B**) 2 h (T2),and (**C**) 5 h (T5). Significantly upregulated and downregulated genes in red and blue, respectively. Numbers in corners indicate significantly differentially expressed genes (DEGs; adjusted *P* < 0.05, |log_2_ fold change| > 1, *n* = 5). (**D**) Principal coordinates analysis (PCoA) based on Euclidean distances of variance-stabilized (VST) transcript counts for *C. butyricum* transcriptomes. PCoA was performed to gain global transcriptional differences over time in MSG-treated samples. Samples clustered clearly by sampling time (T0, T2, T5), indicating temporal shifts in gene expression across growth. Ellipses denote 95% confidence intervals for each time group.

Two hours after MSG treatment, a rapid shift in the overall differentially expressed gene (DEG) profile of *C. butyricum* was observed. MSG induced 124 upregulated and 287 downregulated genes compared to the control. At 5 h post-treatment, a robust response was still evident, with 156 upregulated and 146 downregulated genes in the treated samples relative to the control (Fig. 1A through C). Principal coordinates analysis (PCoA) revealed clear time-dependent transcriptomic trajectories following MSG addition, with samples clustering by time point and the first two axes explaining >50% of the variance (Fig. 1D; PERMANOVA, *P* < 0.05).

**Fig 2:**
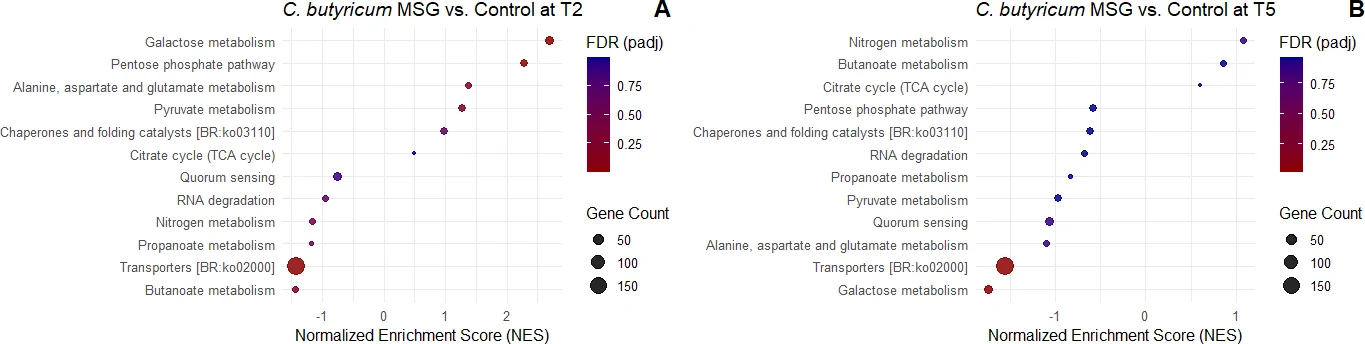
Targeted GSEA of preselected KEGG pathways in *C. butyricum* (MSG vs control). (**A**) Enriched pathways 2 h after treatment (*n* = 5). (**B**) Enriched pathways 5 h after treatment (*n* = 5). Points are positioned by normalized enrichment score (NES; >0 = enriched in MSG, <0 = enriched in CTRL), colored by FDR, and sized by the number of genes contributing to the enrichment (count). Significant enrichment was observed for galactose metabolism, pentose phosphate pathway, and transporters (BR:ko02000) (FDR < 0.05) in both time points. Pathways are listed on the *y*-axis.

Gene set enrichment analysis (GSEA) was performed on KEGG pathways using a ranked list of differentially expressed genes (DEGs) from a predefined targeted gene set. GSEA revealed time-dependent metabolic reprogramming following MSG treatment (Fig. 2A and B). Two hours after MSG exposure, cells showed an activation of central carbon catabolism, including carbohydrate metabolism pathways (galactose metabolism, pyruvate metabolism, and citrate cycle [TCA cycle]), and redox-supporting pathways (including pentose phosphate pathway [PPP]), as well as stimulation of alanine, aspartate, and glutamate metabolism and stress-response genes such as chaperones and folding catalysts. In contrast, pathways related to transport, quorum sensing, nitrogen metabolism, and butanoate metabolism were predominantly transcriptionally repressed. After 5 h, this profile shifted toward a compensatory state. Energy metabolism pathways, including butanoate and nitrogen metabolism, were positively enriched, whereas carbohydrate and glutamate metabolism, as well as chaperone and folding catalyst stress-response, were suppressed, consistent with reduced acute stress signaling and a return toward energy-conserving growth metabolism. Further enzyme-level assignments, including significantly differentially expressed genes for each targeted pathway, are provided in the supplemental material (Fig. S4).

**Fig 3:**
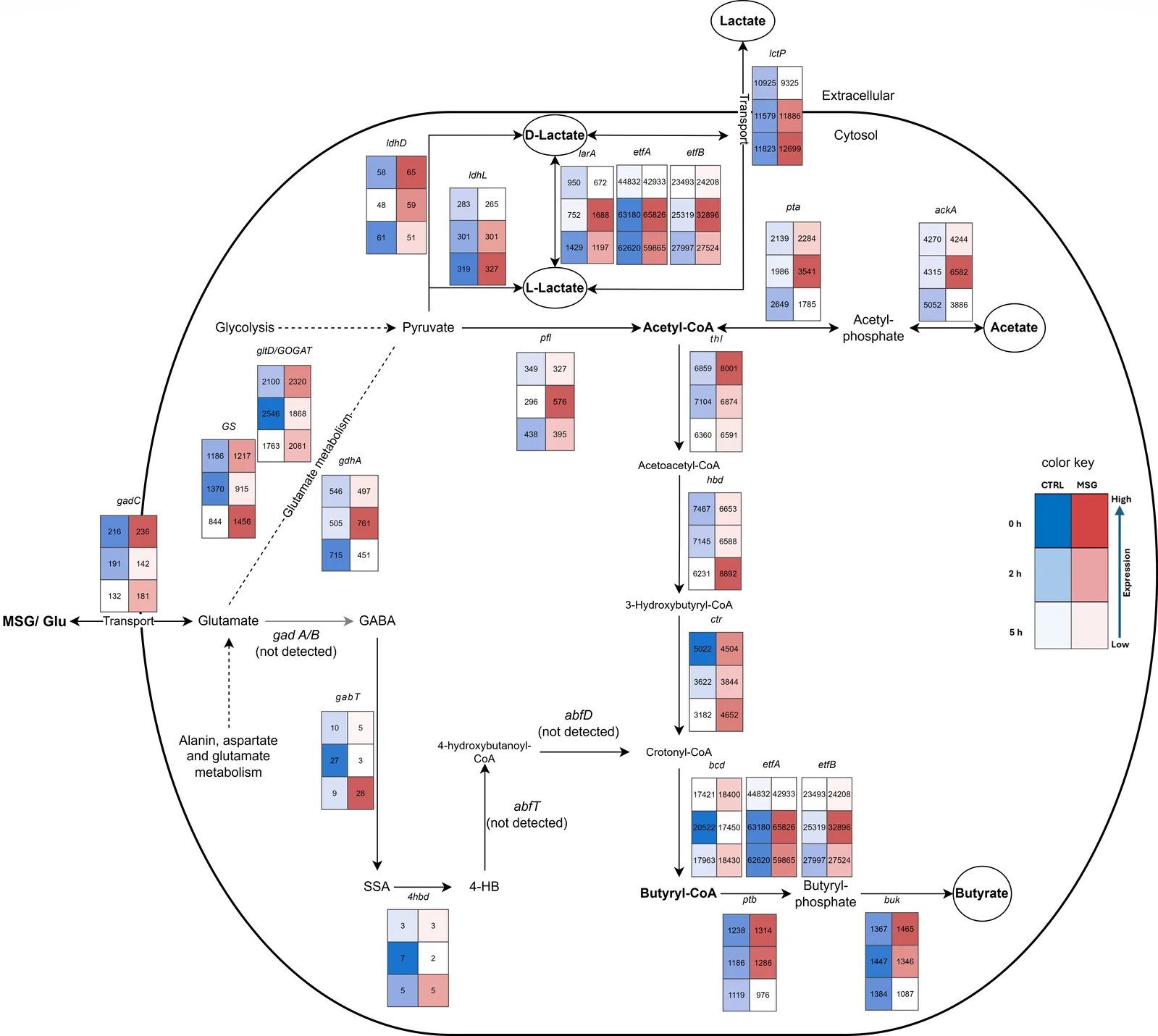
A simplified schematic of central carbon and glutamate-associated metabolism to the major fermentation products (acetate, butyrate, and lactate) in *C. butyricum*. Mini-heatmaps of each gene show mean normalized expression (counts) for CTRL (without MSG; blue column) and MSG-treated (red column) samples across time (0, 2, 5 h). Genes labeled “not detected” were not identified in the data set but are shown to keep the biochemical route complete. Metabolite abbreviations: Glu, glutamate; GABA, γ-aminobutyric acid; SSA, succinate semialdehyde; 4-HB, 4-hydroxybutyrate. Enzyme abbreviations (gene name, enzyme): *ldhL*, L-lactate dehydrogenase; *ldhD*, D-lactate dehydrogenase; *larA*, lactate racemase; *pfl*, pyruvate formate-lyase; *pta*, phosphate acetyltransferase; *ackA*, acetate kinase; *thl*, thiolase/acetyl-CoA acetyltransferase; *hbd*, 3-hydroxybutyryl-CoA dehydrogenase; *crt*, crotonase/enoyl-CoA hydratase; *bcd*, butyryl-CoA dehydrogenase; *ptb*, phosphotransbutyrylase; *buk*, butyrate kinase; *gdhA*, glutamate dehydrogenase; *gltD*, glutamate synthase subunit (GOGAT); *GS*, glutamine synthetase; *gabT*, 4-aminobutyrate aminotransferase (GABA-T); *4hbd*, 4-hydroxybutyrate dehydrogenase; *gadA/B* (not detected), glutamate decarboxylase; *abfT* (not detected), 4-hydroxybutyrate CoA-transferase; *abfD* (not detected), 4-hydroxybutyryl-CoA dehydratase; *etfA*, electron transfer flavoprotein subunit alpha (ETF α-subunit); *etfB*, electron transfer flavoprotein subunit beta (ETF β-subunit).

The expression profiles of genes involved in central carbon and glutamate-associated metabolism and linked to the major fermentation products (acetate, butyrate, and lactate) indicated increased expression of genes supporting conversion of pyruvate-derived acetyl-CoA to the acetate branch (pyruvate formate-lyase [*pfl*], phosphate acetyltransferase [*pta*], and acetate kinase [*ackA*]) in MSG-treated samples after 2 h (Fig. 3), consistent with an early shift toward ATP-generating acetate formation (24). In parallel, the glutamate metabolism gene, glutamate dehydrogenase (*gdhA*), was transcriptionally remodeled and, together with the increased expression of TCA cycle genes at 2 h, suggested transcriptional remodeling consistent with increased routing of carbon through pyruvate-linked metabolism. At 5 h, the expression patterns shifted toward butanoate and energy metabolism, with higher expression across key enzymes in the butyrate branch from acetyl-CoA to butyryl-CoA (3-hydroxybutyryl-CoA dehydrogenase [*hbd*], enoyl-CoA hydratase [*crt*], butyryl-CoA dehydrogenase [*bcd*]). This was accompanied by the induction of the GS-GOGAT cycle (glutamine synthetase and glutamate synthase), consistent with enhanced capacity for high-affinity ammonium assimilation during glutamate-associated nitrogen metabolic remodeling (58). Furthermore, higher induction of a lactate permease (LctP) and an EtfAB-linked lactate utilization module after 5 h was compatible with increased capacity for lactate-linked butyrate formation (27).

#### Modulation of SCFA profile

**Fig 4:**
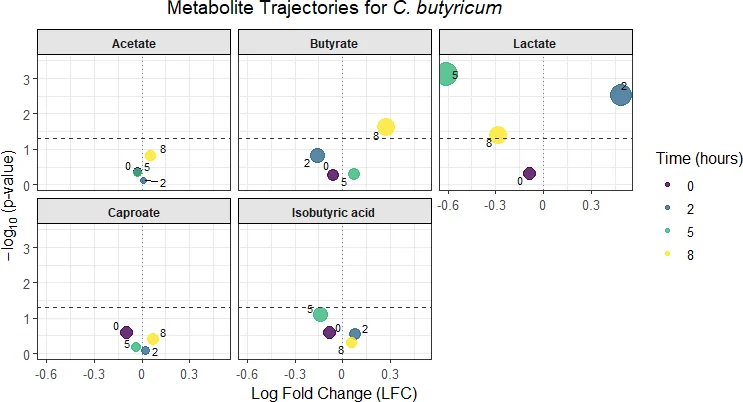
Metabolite trajectories for *C. butyricum* under MSG (*n* = 5). Each panel shows one metabolite. Points (colored/numbered 0, 2, 5, 8 h) give the log fold change (MSG vs CTRL) on the *x*-axis and −log_10_ (*P*-value) on the *y*-axis; the vertical dashed line marks no change (LFC = 0), and the horizontal dashed line marks a nominal significance threshold (*P* = 0.05). Point size indicates the absolute log fold change (|LFC|), reflecting the magnitude of the treatment effect.

The targeted LC-MS/MS analysis indicated the production of acetate, butyrate, caproate, lactate, and isobutyrate in *C. butyricum*. Lactate concentrations increased 1.7-fold at 2 h after MSG treatment, and a significant increase in butyrate concentration with a 1.31-fold (*P* < 0.01) was shown within 8 h after MSG exposure, accompanied by a 24% decrease in lactate (Fig. 4), which was consistent with increased activity of genes associated with the acetyl-CoA-butyrate pathway. Multivariate analysis of SCFA profile in *C. butyricum* showed significant effects of time and MSG, with variance predominantly explained by time (*R*^2^ = 0.21) rather than MSG (*R*^2^ = 0.02). A significant treatment-time interaction (*R*^2^ = 0.27, *P* = 0.004) indicated time-dependent MSG effects rather than a consistent overall shift in metabolite profiles (Table 1). Detailed fixed-effect estimates and pairwise comparisons are documented in Table S4. Extracellular glutamate dynamics with strain-specific post-treatment patterns are provided in Fig. S3.

**TABLE 1:** Permutational multivariate analysis of variance (PERMANOVA) on metabolite production of C. butyricum and B. thetaiotaomicron in the presence of MSG^*a*^

Species	Term	Df	SumOfSqs	*R* ^2^	*F*	Pr (>*F*)
*C. butyricum*	Treatment	1	0.0014	0.0182	1.1461	**0.002**
	Sampling time	3	0.0165	0.2068	4.3472	**0.007**
	Treatment:sampling time	3	0.0213	0.2676	5.6255	**0.004**
*B. thetaiotaomicron*	Treatment	1	0.0004	0.0076	0.3992	**0.011**
	Sampling time	3	0.0171	0.3154	5.5270	**0.004**
	Treatment:sampling time	3	0.0037	0.0682	1.1958	0.364

^*a*^ Significant values are bold.

### Bacteroides thetaiotaomicron

#### Transcriptional reprogramming

**Fig 5:**
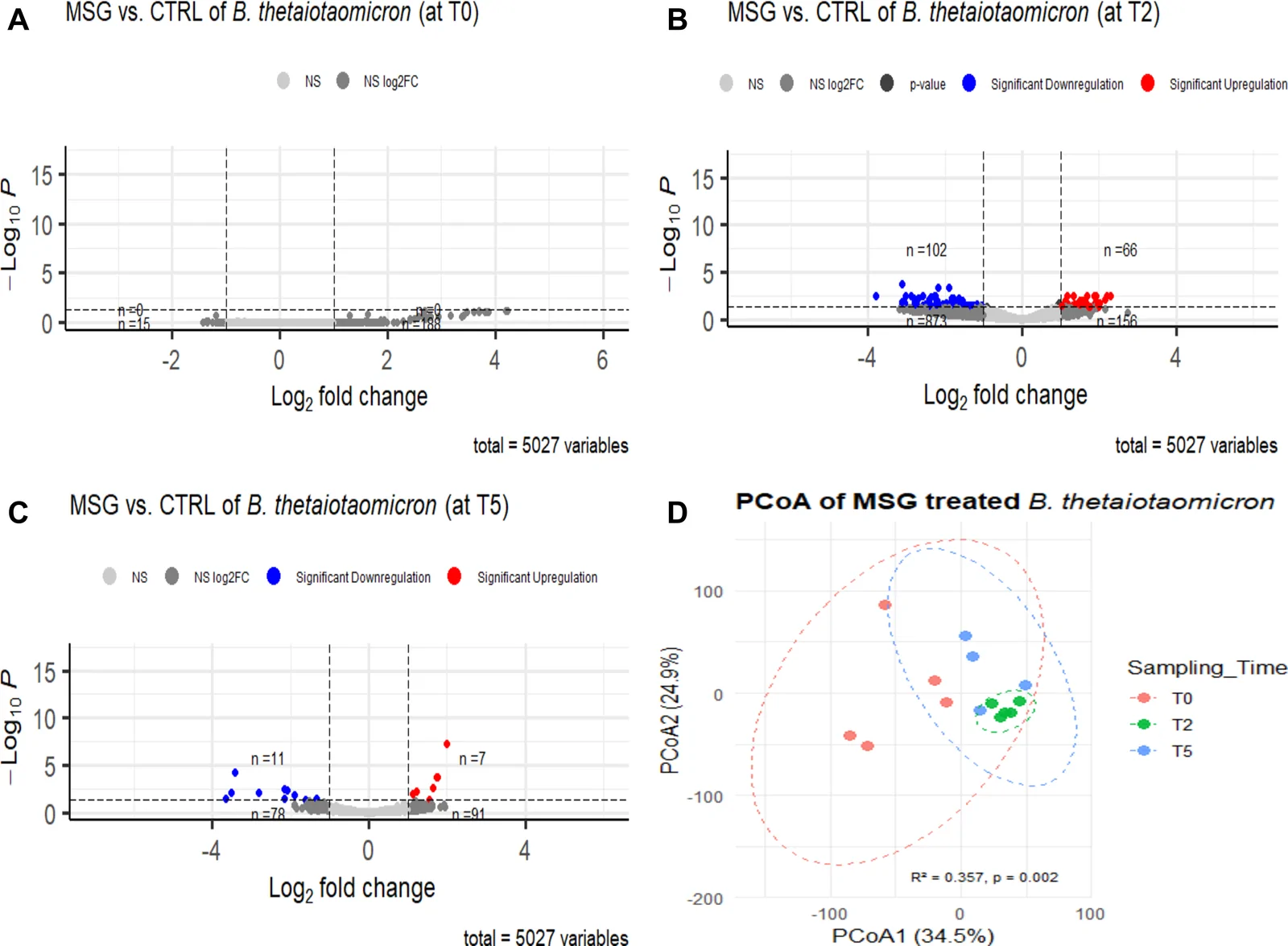
Volcano plots of the differentially expressed genes of *B. thetaiotaomicron* MSG treated vs control at (**A**) baseline (T0), (**B**) 2 h (T2),and (**C**) 5 h (T5). Significantly upregulated and downregulated genes in red and blue, respectively. Numbers in corners indicate significantly differentially expressed genes (DEGs; adjusted *P* < 0.05, |log_2_ fold change| > 1, *n* = 5). (**D**) Principal coordinates analysis (PCoA) based on Euclidean distances of variance-stabilized (VST) transcript counts for *B. thetaiotaomicron* transcriptomes. PCoA was performed to gain global transcriptional differences over time in MSG-treated samples. Samples clustered clearly by sampling time (T0, T2, T5), indicating temporal shifts in gene expression across growth. Ellipses denote 95% confidence intervals for each time group.

The DEG profile of *B. thetaiotaomicron* 2 h after MSG treatment revealed 102 downregulated genes and 66 upregulated genes compared to the control. After 5 h, the transcriptomic response returned largely to baseline, with only 11 downregulated and 7 upregulated genes remaining significantly affected by treatment, aligned with its ability to stabilize metabolism after the initial perturbation (59) (Fig. 5A through C). Additionally, PCoA indicated time-dependent transcriptome shifts following MSG exposure (Fig. 5D). Treated samples were significantly separated by sampling time, with the first principal coordinates accounting for over half of the variance.

**Fig 6:**
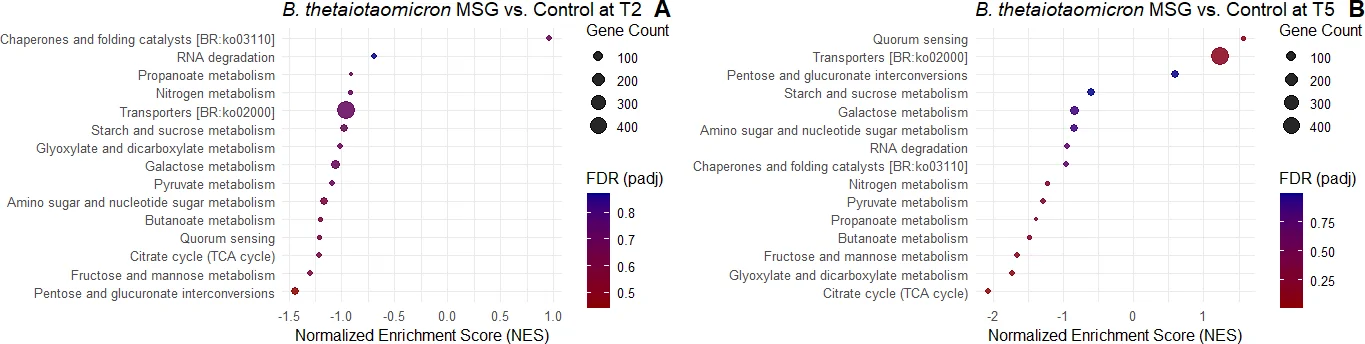
Targeted GSEA of preselected KEGG pathways in *B. thetaiotaomicron* (MSG vs control). (**A**) Enriched pathways 2 h after treatment (*n* = 5). (**B**) Enriched pathways 5 h after treatment (*n* = 5). Points are positioned by normalized enrichment score (NES; >0 = enriched in MSG, <0 = enriched in CTRL), colored by FDR, and sized by the number of genes contributing to the enrichment (Count). Significant enrichment was observed for chaperones and folding catalysts, galactose metabolism, pentose and glucuronate interconversions, amino sugar and nucleotide sugar metabolism, and transporters [BR:ko02000] at 2 h post treatment, and pyruvate metabolism, butanoate metabolism, fructose and mannose metabolism, glyoxylate and dicarboxylate metabolism, and citrate cycle (TCA cycle) after 5 h (FDR < 0.05). Pathways are listed on the *y*-axis.

KEGG-based GSEA of targeted MSG-responsive pathways indicated a time-dependent shift in glycan utilization and stress programs in *B. thetaiotaomicron* (Fig. 6). Two hours after treatment, pathways associated with polysaccharide foraging (starch, fructose, amino sugar metabolism, and pentose interconversions) and broad transporter functions, as well as central energy metabolism (TCA cycle and pyruvate metabolism), were negatively enriched. Following acute proteostasis and stress response, chaperones and folding catalysts were positively enriched. At 5 h, enrichment partially shifted toward increased transport capacity and selective recovery of the glycan-interconversion pathway, while central carbon and energy pathways remained largely negatively enriched. Enzyme-level details for each pathway are provided in the supplemental material (Fig. S5).

**Fig 7:**
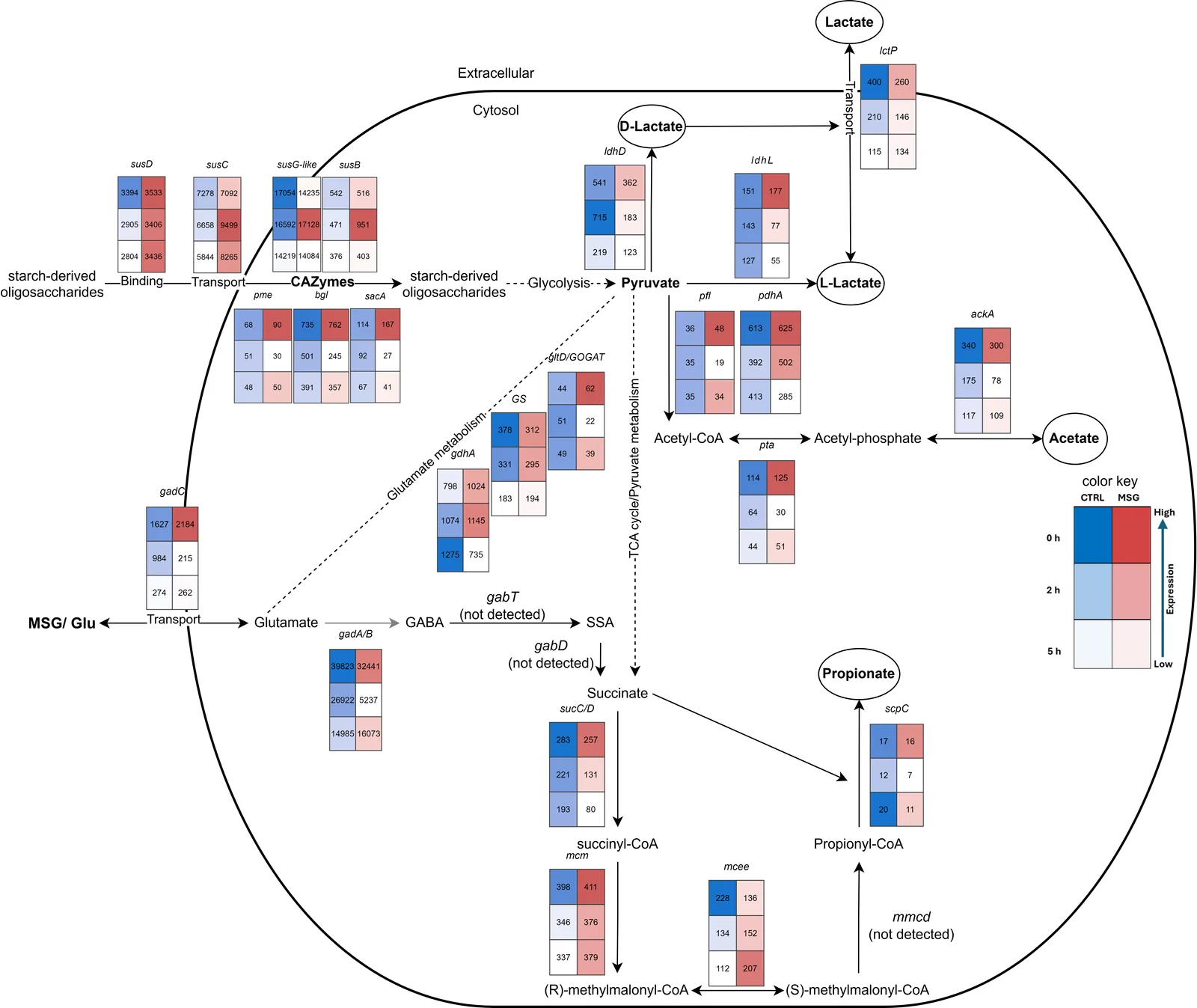
A simplified schematic of starch utilization, central carbon, and glutamate-associated metabolism to the major fermentation products (acetate, propionate, and lactate) in *B. thetaiotaomicron*. Mini-heatmaps of each gene show mean normalized expression (counts) for CTRL (without MSG; blue column) and MSG-treated (red column) samples across time (0, 2, 5 h). Genes labeled “not detected” were not identified in the data set but are shown to keep the biochemical route complete. Metabolite abbreviations: Glu, glutamate; GABA, γ-aminobutyric acid; SSA, succinate semialdehyde. Enzyme/protein abbreviations (gene name, function): *susD*, starch binding protein; *susG*-like, *susG*-like α-amylase; *susB*, glucan 1,4-alpha-glucosidase; *susC*, TonB-dependent transporter; *pme*, pectinesterase, *bgl*, β-glucosidase, *sacA*, β-fructofuranosidase; *lctP*, lactate transporter; *ldhD*, D-lactate dehydrogenase; *ldhL*, L-lactate dehydrogenase; *pfl*, pyruvate formate-lyase; *pdhA*, pyruvate dehydrogenase subunit E1; *pta*, phosphate acetyltransferase; *ackA*, acetate kinase; *gadC*, glutamate/GABA transporter; *gdhA*, glutamate dehydrogenase; *GS*, glutamine synthetase; *gltD*, glutamate synthase (GOGAT); *gadA/B*, glutamate decarboxylase; *gabT* (not detected), 4-aminobutyrate aminotransferase (GABA-T); *gabD* (not detected), succinate semialdehyde dehydrogenase; *sucC/D*, succinyl-CoA synthetase; *mcm*, methylmalonyl-CoA mutase; *mcee*, methylmalonyl-CoA epimerase; *mmcd* (not detected), methylmalonyl-CoA decarboxylase; *scpC*, propionate CoA-transferase.

Analysis of fermentation-associated pathways linked to the major products (Fig. 7) revealed, contrary to expectations (21), no induction of the glutamate-dependent GABA stress response despite excess glutamate from MSG. Instead, the entry enzymes glutamate decarboxylase (GadA/B) and the glutamate/GABA antiporter (GadC) were reduced under MSG at 2 h, and the downstream GABA shunt genes (*gabT* and *gabD*) were not detected. In addition, glutamate synthesis and N-assimilation were reduced, with decreased expression of glutamate synthase (GltD/GOGAT, part of the high-affinity GS-GOGAT pathway) after 2 h, and glutamate dehydrogenase (GdhA, part of the low-affinity NH_4_^+^ assimilation) after 5 h. Despite early negative enrichment of broad transporter and glycan-processing pathways, polysaccharide utilization loci (PULs) showed modular remodeling rather than global repression. Additionally, the reduction of PULs degradative enzymes (pectinesterase [Pme], β-glucosidase [Bgl], and β-fructofuranosidase [SacA]), genes involved in glycan capture and import (TonB-dependent SusC and the starch-binding lipoprotein SusD), and key amylolytic enzymes (SusG-like α-amylase and SusB α-glucosidase) was induced at 2 h and partially recovered by 5 h. For propionate formation via the succinate pathway, succinyl-CoA synthetase enzyme (SucC/D) and the terminal propionate‐releasing step (ScpC) decreased over time and were generally lower under MSG, whereas intermediate isomerization (MceE) showed higher expression, indicating that MSG affects pathway entry/exit control points more than the intermediate conversions. The acetate branch showed mixed regulation, with transcript levels generally declining. Pyruvate formate-lyase (Pfl), phosphate acetyltransferase (Pta), and acetate kinase (AckA) were reduced under MSG, whereas pyruvate dehydrogenase (PdhA) was higher under MSG at 2 h, before dropping by 5 h. Lactate-associated genes, lactate dehydrogenase (*ldhD*/*L*) and lactate permease (*lctP*), were reduced under MSG at 2–5 h, with partial recovery of *lctP* expression at 5 h.

#### Modulation of SCFA profile

**Fig 8:**
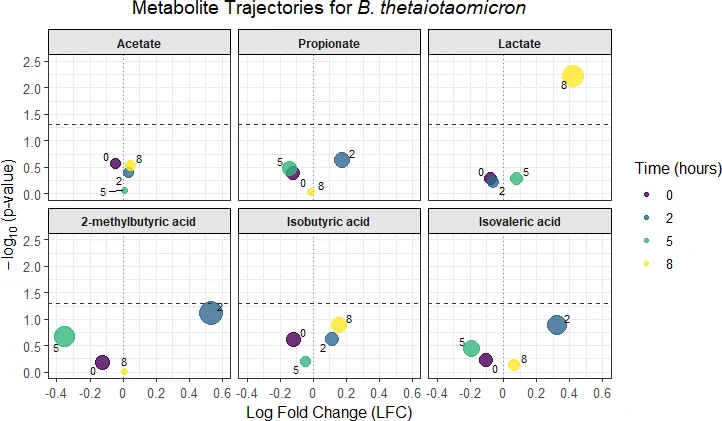
Metabolite trajectories for *B. thetaiotaomicron* under MSG (*n* = 5). Each panel shows one metabolite. Points (colored/numbered 0, 2, 5, 8 h) give the log fold change (MSG vs CTRL) on the *x*-axis and −log_10_ (*P*-value) on the *y*-axis; the vertical dashed line marks no change (LFC = 0), and the horizontal dashed line marks a nominal significance threshold (*P* = 0.05). Point size indicates the absolute log fold change (|LFC|), reflecting the magnitude of the treatment effect.

Targeted metabolomic analysis of *B. thetaiotaomicron* detected acetate, propionate, lactate, isobutyrate, 2-methylbutyrate, and isovalerate (Fig. 8). MSG treatment increased lactate significantly by 53% after 8 h, whereas acetate and propionate (main products) remained stable. In addition, branched-chain fatty acids (2-methylbutyrate, isobutyrate, isovalerate) remained comparable throughout the experiment. Multivariate analysis showed that SCFA profile varied significantly over time, with time explaining most variance (*R*^2^ = 0.32), while MSG contributed a small fraction (*R*^2^ = 0.01), and no significant MSG-time interaction (Table 1). Fixed-effect estimates and Tukey comparisons are provided in Table S4.

## DISCUSSION

The present study demonstrates the impact of monosodium glutamate (MSG), a widely used industrial flavor enhancer that acts as both a nutrient and a potent metabolic signal, eliciting taxon-specific regulatory responses in gut bacteria. Integrating dynamic transcriptomic and metabolomic analyses of two prominent human intestinal bacteria, *Clostridium butyricum* and *Bacteroides thetaiotaomicron,* revealed distinct physiological response strategies, supporting the use of MSG as a model compound to study nutrient signaling and interspecies metabolic diversity in the gut ecosystem.

Glutamate occupies a uniquely integrative position in both microbial and host metabolism, functioning as a carbon skeleton and a redox-active intermediate (17, 19). It plays a crucial role in regulating bacterial nitrogen by serving as the donor and acceptor of the central amino group and enhances growth and carbon flux, while simultaneously inhibiting nitrogen accumulation (24, 60). In the intestinal lumen, it is metabolized by host epithelial cells and by gut microorganisms, providing a bidirectional conduit between diet and physiology (1, 17, 61).

In *Clostridium butyricum*, exposure to MSG triggered rapid activation of pathways regulating central carbon metabolism by shifting toward the activation of carbohydrate-catabolic and ATP-generating pathways and induced specific sugar uptake by shunting carbon into the pentose phosphate pathway (NADPH/ribose). This broad activation pattern indicates that glutamate is readily incorporated into central metabolism and acts as a regulatory signal that enhances redox-associated pathways rather than a simple supplemental nutrient. This agrees with models linking glutamate to carbon-nitrogen metabolism and redox balance, but not to the canonical GadA/B-GadC acid resistance mechanism, as *gadA/B* was not detected (20, 24). Another major early effect was the broad restriction of import and export by downregulating transporters to prevent an uncontrolled influx of solutes in response to the modest ionic perturbation from excess MSG (22). At a later time point (5 h), the upregulation of genes within the GABA shunt modules suggested that exogenous glutamate, as a nitrogen-rich molecule and a potential carbon skeleton feeder, promoted energy generation via the glutamate-GABA-butyrate axis, consistent with potential NAD^+^ regeneration and a capacity for reinforcement of redox balance while routing carbon toward the butyrate branch, accompanied by a marked increase in butyrate production (58, 62), while acetate biosynthetic pathway enzymes were repressed, potentially due to the depletion of available sugar and ammonium, indicating a lower glycolytic and acetyl-CoA flux. This network is biochemically linked to substrate-level phosphorylation, proton-consuming reactions, and redox cofactor turnover, suggesting a potential role in stabilizing intracellular pH and redox homeostasis, and ensuring cell survival in the intestinal acidic environment (63). Consistently, extracellular glutamate declined after the initial MSG-associated increase, supporting glutamate turnover during the butyrate-associated response. Such coordination between catabolic and protective pathways suggested that *C. butyricum* perceives exogenous glutamate as a metabolic opportunity, optimizing both energy yield and redox poise, supporting C. *butyricum’s* metabolic strategy as a specialist in fermenting amino acids and carbohydrates (62). Comparable stimulation of butyrate synthesis by amino acid supplementation has been observed in other *Clostridium* species (glutamate to glutarate or 4-aminobutyrate/GABA) (28, 62), supporting the idea that glutamate serves as an anaplerotic hub, linking amino acid catabolism to SCFA biosynthesis through shared intermediates such as succinyl-CoA and acetyl-CoA. Along with expression of genes involved in redox cofactor turnover (e.g., NADPH-dependent glutamate synthase and ferredoxin-dependent nitrite reduction), *C. butyricum* entered a metabolically active, butyrogenic state. This integrated response is consistent with a regulatory feed-forward mechanism, in which transcriptional upregulation of glutamate catabolism was associated with increased expression of butanoate-pathway genes, allowing the bacterium to potentially exploit glutamate-derived carbon. This suggested that *C. butyricum* coordinated glutamate-associated carbon metabolism with redox-related pathways, linking energy-associated metabolism to the balance between lactate utilization and butyrate production, resulting in a metabolically active but self-regulating state that maximizes energetic efficiency under changing nutrient conditions. While the present transcriptome-metabolite integration supports a compensatory metabolic shift, future studies combining ^13^C-based fluxomics with targeted NAD^+^/NADH and NADP^+^/NADPH measurements would further resolve the underlying carbon-routing and redox mechanisms.

On the other hand, *Bacteroides thetaiotaomicron* appears to interpret MSG exposure as a signal of environmental fluctuation, leading to short-term reallocation of cellular resources and adjustment of osmotic and ionic balance to maintain homeostasis, rather than a clear shift toward exploiting glutamate as an energy source. This contrasts with studies which report that intestinal *Bacteroides* use glutamate- or glutamine-derived GABA for pH-regulated acid stress tolerance (21). Here, *gadA/B* and downstream GABA-shunt genes were not induced, suggesting a transient, phase-dependent homeostatic response rather than absent glutamate responsiveness. After 2 h, MSG modulated a membrane transport system by impacting transport and envelope functions. In parallel, glycan foraging was transiently reprogrammed. Carbohydrate and glycan-processing pathways, along with multiple PUL-associated degradative enzymes, indicated transient repression, even though key components of starch- and sucrose-related PULs (SusC/D/B/G) were induced, pointing to a metabolic prioritization mechanism and modular remodeling. This pattern fits with a pause-and-rebalance response and a strategy of metabolic conservation (64). The subsequent partial recovery of gene expression after 5 h indicates that this response is reversible and tightly regulated, a hallmark of the organism’s hierarchical control of its PUL network (29, 30, 65). Together with the extracellular glutamate dynamics (Fig. S3), this suggests that a return toward baseline can be best explained as a combined effect of reduced glutamate availability and homeostatic adaptation, rather than adaptation alone. Such a buffering mechanism allows *B. thetaiotaomicron* to maintain metabolic stability under transient nutrient perturbations, minimizing metabolic noise and protecting its ecological niche within the gut consortium (65, 66). The stable SCFA profile observed across treatments further indicated that *B. thetaiotaomicron* sustains a steady-state fermentation output, decoupling transient transcriptional fluctuations from core metabolic fluxes. Such metabolic buffering aligns with its ecological role as a glycan generalist and stabilizer species, capable of adjusting to diverse substrate inputs without disrupting community-level function (67). These divergent strategies underscore the functional heterogeneity of the gut microbiota, with glutamate driving strain-specific responses that can create selective metabolic niches.

From an ecological perspective, *C. butyricum* might act as a metabolic activator, converting exogenous amino acids into energetically favorable SCFA. *B. thetaiotaomicron* likely functions as a system stabilizer, modulating its transcriptional programs to maintain resource efficiency. The coexistence of such divergent behaviors contributes to the resilience and homeostasis of the intestinal ecosystem under nutrient perturbation and may explain the inconsistent outcomes of MSG supplementation studies (25, 26), where bulk analyses overlook species-specific functional responses masked within the aggregate community.

The metabolic responses to MSG and the differential SCFA production patterns, especially the increase in butyrate levels observed in *C. butyricum*, could have dose-dependent health consequences for the host. Butyrate serves as an energy source for colonocytes, a regulator of epithelial barrier function, and as a histone deacetylase inhibitor, modulating inflammatory pathways and glucose homeostasis (68, 69). Butyrate insufficiency has been linked to insulin resistance, type 2 diabetes, and cardiovascular disease (70), while excessively high butyrate levels could suppress epithelial barrier integrity or stem cell production (71). Although the Bacillota-Bacteroidota ratio can vary substantially among healthy individuals as a result of dietary, environmental, and physiological factors, it is generally stable within a given person and is often considered a hallmark of a balanced gut ecosystem (72). Shifts toward Bacillota dominance have been associated with increased energy harvest from ingested food and, in some studies, with obesity-linked metabolic phenotypes (73, 74). In contrast, a shift toward Bacteroidota dominance could reflect greater reliance on polysaccharide degradation and may correspond with leanness, lower fermentation capacity, or altered bile acid metabolism (4).

Beyond the immediate importance of MSG, these findings provide a framework for understanding dietary amino acids as ecosystem-level regulatory molecules. By linking carbon, nitrogen, and redox metabolism across distinct microbial guilds, glutamate signaling emerges as a key component of the gut’s chemical communication network, a process that may shape both microbial ecology and host metabolic health.

In this study, glutamate-driven metabolic activities were tested using a universal growth medium, enabling direct comparison of MSG responses while minimizing strain-specific medium optimization effects. As media components can influence bacterial metabolism (75), future studies are needed to ascertain whether the observed molecular responses to glutamate are conserved across different nutritional environments. Furthermore, the physiological relevance of luminal MSG exposure is region-dependent (76). As the strains studied here are primarily large-intestine-associated anaerobes, our findings mainly inform colonic microbial responses and should not be directly extrapolated to the small intestine, which differs in nutrient sensing, absorption, transit, and environmental conditions (77). A limitation of this study is that qPCR estimates total genome-equivalent biomass and does not directly distinguish viable cells from recently dead or damaged cells. This limitation is partly reduced by the short-term pure-culture design and the use of strain-specific TaqMan assays, but DNA from recently lysed cells may still contribute to the qPCR signal. In addition, OD_600_ provided an independent biomass-related readout and confirmed stationary-phase conditions at the time of MSG exposure, but OD_600_ also does not resolve viability and can be affected by cell morphology, aggregation, or medium turbidity. Future studies should, therefore, complement qPCR and OD_600_ measurements with CFU enumeration, viability staining-based flow cytometry, or PMA-qPCR to directly distinguish viable, viable but non-culturable, and dead-cell fractions.

### Conclusion

This research highlights the importance of combining transcriptomic and metabolomic data to understand microbial nutrient signaling. It demonstrates that short-term dietary inputs can trigger rapid reprogramming and adaptation even in isolated species, with distinct response patterns observed in the two strains studied from different phyla. In conclusion, MSG serves as an effective probe of microbial metabolic signaling. Its distinct effects on *C. butyricum* and *B. thetaiotaomicron* illustrate the diversity of bacterial adaptation strategies in the gut ecosystem. By acting as both a nutrient and signal, glutamate differentially modulates energy flow and redox homeostasis, with potential implications for community stability. These results clarify the molecular foundations of microbial responses to dietary additives and open new perspectives for manipulating gut metabolism through targeted nutritional interventions. Future work should include additional strains to determine which responses are conserved vs strain-dependent and extend these findings to multispecies consortia and *in vivo* systems, where host-microbe and microbe-microbe interactions further shape metabolic signaling. To better capture potential synergistic effects, studies incorporating more realistic dietary conditions, including mixtures of flavors and flavor enhancers, would provide valuable insights.

## Data Availability

The raw Illumina NextSeq 550 cDNA RNA-seq reads generated in this study are available in the NCBI Sequence Read Archive (SRA) under BioProject accession number PRJNA1481563 and SRA Study accession number SRP712216.

## SUPPLEMENTAL MATERIAL

Supplemental material msphere.00190-26-s0001.docx Supplemental methods and results, Fig. S1 to S5, and Tables S1 to S4.

ASM does not own the copyrights to Supplemental Material that may be linked to, or accessed through, an article. The authors have granted ASM a non-exclusive, world-wide license to publish the Supplemental Material files. Please contact the corresponding author directly for reuse.
