# Supplementary material for: In vitro metabolic signaling in two intestinal bacterial isolates: glutamate-driven transcriptional and functional reprogramming in Clostridium butyricum and Bacteroides thetaiotaomicron
Source: mSphere. 2026 Aug 10;11(8):e00190-26. doi: 10.1128/msphere.00190-26 (PMC13508794; doi:10.1128/msphere.00190-26)
Supplement: Supplementary file 1 [file msphere.00190-26-s0001.docx]

# Supplementary

## Methods

**Growth-Curve Experiment:** To characterize the growth dynamics of each strain under the defined culture conditions, a preliminary growth-curve experiment was performed. Inocula for both strains were standardized to ~10^8^ cells per 100 mL culture, based on flow cytometric cell counts, and incubated at 37 °C without stirring under strict anaerobic conditions (n = 3). OD_600_ was measured hourly for 42 hours by aseptically sampling cultures into a sealed microplate and immediately measuring absorbance using a BioTek Epoch 2 microplate spectrophotometer (Agilent Technologies, Santa Clara, CA, USA).

**Flow Cytometry:** Absolute cell numbers of both strains were quantified by flow cytometry (BD FACSCanto™ II, BD Biosciences) using SYTO™ 9 (Thermo Fisher Scientific, Waltham, MA, USA) staining and BD Trucount™ tubes (BD Biosciences). Cell numbers were calculated in FlowJo v10.9.0 based on bead-normalized counts and used to standardize inoculum, which indicated that an OD_600_ of 1.0 for *C. butyricum* corresponds to approximately 3.38E+09 cells mL^-1^ and an OD_600_ of 1.0 for *B. thetaiotaomicron* to 2.23E+10 cells mL^-1^.

**qPCR Reaction Preparation:** Reactions (20 µL) were prepared with 10 µL TaqMan™ Fast Advanced Master Mix (Thermo Fisher Scientific, Darmstadt, Germany), 0.4 µL of each primer, 0.25 µL probe, 6.95 µL nuclease-free water, and 2 µL diluted DNA template (4-10 ng per reaction). Each probe carried a FAM reporter label at the 5’-end and a 3’-TAM quencher label.

Table S1 Primer and probe sequences of C. butyricum and B. thetaiotaomicron used in qPCR

| **Bacteria** | **Primer and probe (5’-3’)** | **Target gene** | **Amplicon size** |
| --- | --- | --- | --- |
| ***C. butyricum*** | F: CAGCCTTGAACAAGGATAGGA  R: ATAACTGCCCGTATCAAGACC  P: TGGAGCATTTAAGGGAGAAGCTTACAGA | *yaaA* | 113 bp |
| ***B. thetaiotaomicron*** | F: GGCGACATGGGTGACAATAA  R: CACGGAGCTGGTTGATAAAGA  P: TAGGTTTGCAGGCACGTCTGATGT | *recA* | 121 bp |

F, forward primer; R, reverse primer; P, probe.

**Quantitative Real-Time PCR Conditions**: Reactions were run on an Applied Biosystems 7300 Real-Time PCR System (Thermo Fisher Scientific, Darmstadt, Germany), including an initial hold at 50 °C for 2 min, followed by a 5 min hold at 95 °C and 40 cycles of denaturation at 95 °C for 30 s and annealing/extension at 60 °C for 45 s.

**Comparative Genome Analysis:** To compare the genomic potential of the two gut bacteria, we performed a two-genome comparative analysis using anvi’o (V8). The reference genomes were retrieved from the NCBI RefSeq database (*B. thetaiotaomicron* DSM 2079-ASM15132v1; *C. butyricum* DSM 10702-ASM1413179v1). Gene prediction and functional annotation were conducted using anvi’o with NCBI COG and KEGG KOfam databases, and genome similarity was assessed by average nucleotide identity (ANI).

**Metabolite Profiling Following 3-NPH Derivatization (55):** Chromatographic separation was performed on a Kinetex C18 column (100 × 2.1 mm, 100 Å, 1.7 µm; Phenomenex) using a water/acetonitrile (0.1% formic acid) gradient from 23% to 100% B over 7 min (total run time 8.5 min); and analytes were measured on a Nexera LC-40 UHPLC system (Shimadzu) coupled to a QTRAP 6500+ triple quadrupole mass spectrometer (Sciex, Darmstadt, Germany) with source conditions (500 °C, curtain gas 35 psi, ion source gas 1/2 55/65 psi, -4500 V) and MRM transitions optimized using commercial SCFA and bile acid standards.

## Results

**Growth Curves of Control and MSG-Treated Conditions**

**A**

**B**

Figure S1 Growth-phase determination and post-treatment biomass dynamics. A) Preliminary OD_600_ growth curves of C. butyricum and B. thetaiotaomicron under untreated anaerobic culture conditions over 42 hours. The red arrow indicates the 18-hour time point used for MSG addition in the main experiment. Error bars indicate standard deviation from three biological replicates. B) OD_600_ trajectories of control and MSG-treated cultures during the main experiment, shown from 0 to 26 hours of incubation. T0, T2, T5, and T8 correspond to before MSG addition, 2 hours, 5 hours, and 8 hours after MSG addition, respectively. Error bars indicate standard deviation from five biological replicates.

Table S2 Linear mixed-effects analysis of total bacterial biomass dynamics in C. butyricum and B. thetaiotaomicron with and without MSG supplementation based on qPCR quantification of DNA copies.

| **Species** | **Term** | **SumOfSqs** | **MeanOfSqs** | **Df** | **F** | **Pr ( > F)** |
| --- | --- | --- | --- | --- | --- | --- |
| ***C. butyricum*** | Sampling time | 0.0058 | 1.9315e-03 | 3 | 0.1585 | 0.9227 |
|  | Treatment | 0.02616 | 2.6163e-02 | 1 | 2.1464 | 0.1623 |
|  | Treatment: Sampling time | 0.0009 | 2.9637e-04 | 3 | 0.0243 | 0.9947 |
| ***B. thetaiotaomicron*** | Sampling time | 0.0016 | 5.2578e-04 | 3 | 0.0636 | 0.9783 |
|  | Treatment | 0.0287 | 2.8693e-02 | 1 | 3.4733 | 0.0808 |
|  | Treatment: Sampling time | 0.0003 | 9.9249e-05 | 3 | 0.0120 | 0.9981 |

**Comparative Genome Analysis:** Given that the two organisms originate from different phyla, their genomic relatedness was assessed to justify strain-resolved downstream analyses (Fig. S2). This allowed us to determine whether condition-specific responses reflected a lack of gene expression or the true absence of the gene within a strain’s genome. Self-comparisons showed ANI and alignment percentage (AP) values > 99%, whereas cross-species comparisons between *B. thetaiotaomicron* DSM 2079 and *C. butyricum* DSM 10702 showed ANI = 0.0 and AP = 0%, confirming that no meaningful nucleotide-level similarity was detected under the applied ANI settings (Table S3). The *B. thetaiotaomicron* genome was 6.29 Mb in 2 contigs, with 4,964 predicted protein-coding genes and a GC content of 42.9%, whereas the *C. butyricum* genome was 4.71 Mb in 4 contigs, with 4,301 genes and a GC content of 28.7%. The two-genome comparative analysis comprised 8,378 gene clusters, of which only 136 (1.6%) constituted the core genome (shared by both strains), while 8,242 (98.4%) were strain-specific accessory clusters. This strong divergence justified a separate, strain-specific interpretation of transcriptomic and metabolomic responses to MSG.

Table S3 Average nucleotide identity (ANI) and alignment percentage (AP) between the genomes used in this study.

| **Query** | **Reference** | **ANI** | **AP** |
| --- | --- | --- | --- |
| B. thetaiotaomicron DSM 2079 | B. thetaiotaomicron DSM 2079 | > 99 | > 99% |
| C. butyricum DSM 10702 | C. butyricum DSM 10702 | > 99 | > 99% |
| B. thetaiotaomicron DSM 2079 | C. butyricum DSM 10702 | 0.0 | 0% |
| C. butyricum DSM 10702 | B. thetaiotaomicron DSM 2079 | 0.0 | 0% |

Figure S2 Comparative genome structure of Clostridium butyricum DSM 10702 and Bacteroides thetaiotaomicron DSM 2079. The circular anvi’o visualization summarizes shared and strain-specific gene clusters between the two genomes. Core and cloud gene clusters are indicated to show the limited shared gene-cluster fraction and the large strain-specific accessory genome structure. Detailed ANI and alignment percentage values are provided in Table S3.

**Extracellular Glutamate Dynamics Following MSG Exposure**

Figure S3 Extracellular glutamate dynamics following MSG exposure. Extracellular glutamate concentrations were measured by LC-MS/MS over time in Clostridium butyricum and Bacteroides thetaiotaomicron cultures grown with and without MSG. Boxplots show the distribution of measured extracellular glutamate concentrations for each treatment and sampling time, with individual points representing biological replicates. Black diamonds indicate linear mixed-effects model estimated marginal means. The model included treatment, sampling time, and their interaction as fixed effects, and an AR(1) correlation structure to account for repeated measurements over time. Asterisks indicate significant time-specific pairwise treatment contrasts between MSG-treated and control cultures based on raw LME-derived p-values and are shown for descriptive visualization. Time point 0 indicates the sampling point immediately before MSG addition in treated cultures and the corresponding baseline sampling point in control cultures; subsequent time points represent the duration of exposure after MSG supplementation. Glutamate measurements were available for three biological replicates and were therefore interpreted as supportive evidence for extracellular substrate dynamics rather than as standalone proof of carbon flux.

**Linear mixed-effects modeling results *of* metabolite production**

Table S4 Linear mixed-effects modeling results for significant shifts (p < 0.05) in metabolite production via one fixed factor in ANOVA analysis. Significant values (p < 0.05) are bold; “-” indicates not detected.

| **Metabolites** | ***Clostridium butyricum*** | | | ***Bacteroides thetaiotaomicron*** | | |
| --- | --- | --- | --- | --- | --- | --- |
|  | Treatment | Sampling Time | Treatment: Sampling Time | Treatment | Sampling Time | Treatment: Sampling Time |
| **Acetate** | 0.41 | 0.62 | 0.26 | 0.23 | **0.001** | 0.35 |
| **Butyrate** | 0.55 | 0.18 | **0.01** | - | - | - |
| **Propionate** | - | - | - | 0.4 | **< 0.001** | 0.28 |
| **Caproate** | 0.22 | 0.43 | 0.47 | - | - | - |
| **Lactate** | 0.45 | **< 0.001** | **< 0.001** | 0.5 | 0.38 | **0.003** |
| **Isobutyrate** | 0.22 | **0.04** | 0.07 | 0.2 | **< 0.001** | **0.01** |
| **Isovalerate** | - | - | - | 0.6 | **< 0.001** | 0.19 |
| **2-methylbutyrate** | - | - | - | 0.6 | **< 0.001** | 0.05 |

**Targeted DEG**

Figure S4 Heatmap of the targeted DEG of C. butyricum (n = 5). Columns represent time points (T0 = before treatment, T2 = 2 hours, T5 = 5 hours). Rows are annotated with functions. Colors indicate log_2_ fold change (case vs. control). Pathway and subclass categories are annotated. Statistical significance is shown with asterisks (* p < 0.05, ** p < 0.01, *** p < 0.001).

Figure S5 Heatmap of the targeted DEG of B. thetaiotaomicron (n = 5). Columns represent time points (T0 = before treatment, T2 = 2 hours, T5 = 5 hours). Rows are annotated with functions. Colors indicate log_2_ fold change (case vs. control). Pathway and subclass categories are annotated. Statistical significance is shown with asterisks (* p < 0.05, ** p < 0.01, *** p < 0.001).
